# Sustainable Intensification with Cereal-Legume Intercropping in Eastern and Southern Africa

**DOI:** 10.3390/su11102891

**Published:** 2019-05-21

**Authors:** Abednego Kiwia, David Kimani, Rebbie Harawa, Bashir Jama, Gudeta W. Sileshi

**Affiliations:** 1 Alliance for Green Revolution in Africa (AGRA), West End Towers, Waiyaki Way, P.O. Box 66773 Westlands, Nairobi 00800, Kenya; AKiwia@agra.org (A.K.); DKimani@agra.org (D.K.); RHarawa@agra.org (R.H.); 2 Islamic Development Bank, 8111 King Khalid St., Al Nuzlah Al Yamania Dist., Jeddah 22332-2444, Saudi Arabia; BAdan@isdb.org; 3 Plot 1244, Ibex Hill, 10100 Lusaka, Zambia; 4 School of Agricultural, Earth and Environmental Sciences, University of KwaZulu-Natal, Pietermaritzburg 4041, South Africa

**Keywords:** agronomic efficiency, climate-smart agriculture, mother-baby trial, nutrient mining, trade-off

## Abstract

Much research has been conducted on cereal-legume intercropping as a sustainable intensification (SI) practice in Eastern and Southern Africa (ESA). However, the role of inorganic fertilizers in sustainably intensifying intercropping systems has not been systematically analyzed. Therefore, the objective of the present analysis was to assess the role of inorganic fertilizer use in cereal-pigeonpea (*Cajanus cajan*) intercropping in terms of SI indicators, namely, yield, production risks, input use efficiency, and economic returns. The data used for this analysis were gathered from over 900 on-farm trials across Kenya, Tanzania, and Mozambique. All SI indicators assessed showed that intercropping combined with application of small amounts of inorganic fertilizers is superior to unfertilized intercrops. Fertilizer application in the intercropping system improved cereal yields by 71–282% and pigeon pea yields by 32–449%, increased benefit–cost ratios by 10–40%, and reduced variability in cereal yields by 40–56% and pigeonpea yields by 5–52% compared with unfertilized intercrops. Improved yields and reduced variability imply lowering farmers’ risk exposure and improved credit rating, which could enhance access to farm inputs. We conclude that the strategic application of small amounts of inorganic fertilizers is essential for the productivity and economic sustainability of cereal-pigeonpea intercropping under smallholder farming in ESA.

## 1. Introduction

The smallholder agricultural sector in Eastern and Southern Africa (ESA) region is heavily constrainedbydecliningpercapitalandholding[1],lossofsoilfertility[2,3],andclimatevariability[4–6]. Asinmostofsub-SaharanAfrica, thisregionisseverelyaffectedbylanddegradation[7]. Severaldecades of farming without use of fertilizer inputs, from both organic and inorganic sources, has stripped the soils of the vital nutrients needed to support plant growth [2], and this is now posing unprecedented social, economic, and environmental problems [7]. Sustainable intensification (SI) of smallholder agriculture has been recognized as a crucial component of the strategy towards reversing the trend in land degradation and increasing food production [1,8,9]. Sustainable intensification (SI) is defined as producing more output from the same area of land while reducing the negative environmental impacts, and at the same time increasing contributions to natural capital and the flow of environmental services [9,10]. SI is now recognized as one of the cornerstones of climate-smart agriculture (CSA), i.e., agriculture that achieves the triple objectives of increasing productivity, adaptation to climate change, and mitigating greenhouse gas emissions [11].

Cereal-legume intercropping has been recognized as one of the SI pathways because intercropping provides greater stability than sole cropping (monocultures) in terms of soil fertility improvement, yields enhancement, and financial returns [12,13]. Cereal-legume intercropping also ensures diversification of diets and risk reduction in the case of failure of one of the crops [12]. Among the legumes, *Cajanus cajan* (L.) Millsp (pigeonpea) has been shown to hold the greatest potential for intercropping with cereals in ESA because of its drought-tolerance ability and the role it plays in household nutrition, income, and enhancing productivity. Unlike most legumes, pigeonpea is well adapted to semi-arid and arid regions, and is traditionally intercropped with maize (*Zea mays* (L.)), sorghum (*Sorghum bicolar* (L.) Moench), and millet (*Eleucine coracana* (L.) Gaertn, ssp. *Coracana*) in ESA. For example, the Konso people, living in an arid pastoral area of south-central Ethiopia, have for centuries practiced pigeonpea intercropping with ratooned sorghum [14]. Similarly, in southern Malawi pigeonpea is traditionally ratooned and managed as a perennial crop in maize cropping systems [15]. The intercrop diversifies the production system and helps to minimize risks associated with growing only one crop [8]. Additionally, it can provide opportunities for increased use efficiency of the production resources, including water, and is thus a sound cropping system in drylands.

In areas prone to extreme weather conditions, intercropping of staple cereals with pigeonpea has been reported to provide greater insurance against crop failure [8,16,17]. In addition, most pigeonpea varieties mature during the dry season long after farmers have harvested their cereal crops, thereby bridging the hunger period and providing food security to rural households [18–20]. It also provides additional benefits, such as fodder for livestock and firewood for household energy needs [19,21]. The firewood supply can have labor-saving benefits for women who are traditionally expected to perform this task for the family. There could also be environmental benefits resulting from reduced pressure for firewood on the woodlands, even if only for short periods.

Due to its complementarities with cereals and its ability to fix nitrogen (N) biologically from the atmosphere, the benefits of cereal-pigeonpea intercropping in long-term soil fertility improvement have been well documented [16–18]. Across the semi-arid areas of ESA, pigeonpea is cultivated on over 400,000 ha [22], but the potential is much greater. The rising and sustained import demand from India and the Middle East [22,23] is likely to increase the land area that could potentially be brought under the crop. The challenge to improved trade prospects has, however, been its low productivity, especially under smallholder cropping systems, which is the dominant form of pigeonpea production [22]. Pigeonpea yields are typically under 1.0 t ha^−1^ in these systems [22,23]. The yields could, however, be more than tripled with the use of improved varieties and good agronomic practices, including the use of small amounts of fertilizers or manure [24].

Much research has been done on cereal-pigeonpea intercropping on research stations, comparing yields in intercrops with sole maize [18,25,26]. Most of the studies examined the primary trade-offs in cereal-pigeonpea intercropping, which include competition [20,26,27] and nutrient mining [19]. For example, in a long-term study in Zimbabwe [26], maize yields without fertilizer were lower in the intercrop than the sole maize in five out of 12 seasons. Results from other studies conducted through a multi-location trials approach in Tanzania and Malawi raise concerns about the long-term sustainability of the production system based on the levels of nutrients removed with crop harvests [19]. The annual net removals of N and P were estimated at 31–49 kg ha^−1^ in Malawi and 35–68 kg ha^−1^ in Tanzania. For P, the estimates stood at 9–17 kg ha^−1^ and 6–25 kg ha^−1^, respectively.

Several studies, including one that conducted meta-analysis of several on-farm trials [12], show that cereal yields are generally less than 3.0 t ha^−1^, and that a global consensus is building around the minimum threshold required to achieve household food self-sufficiency and kick-start a smallholder-led “green revolution” in Africa [2]. This is particularly important for maize, which is a staple food crop in many countries in sub-Saharan Africa (SSA). Raising the yields is important given that when farmers move from under 1.0 to 3.0 t ha^−1^ or more, they generally begin to diversify their production to income-generating practices that also engender the sustainability of the production system [2]. This level of productivity cannot be achieved under smallholder farm conditions unless external inputs, such as fertilizer and manure, are applied. However, the role of inorganic fertilizer in sustainably increasing productivity in intercropping has not been systematically studied. Information is also virtually lacking on the profitability of fertilizer use in cereal-pigeonpea intercropping systems in much of the ESA.

Increasing fertilizer use in legume intercrops would require awareness creation, given that the practice is not common among smallholder farmers in the ESA region [27,28]. To this end, the Alliance for Green Revolution in Africa (AGRA), through its Soil Health Program, supported several projects across SSA. This involved establishing a large number of participatory trials on farmers’ fields over 4–5 years, which allowed farmers to participate actively in their design and management. In addition to demonstrating technologies, the trials were used to generate yield and socio-economic data that could be used to assess the performance of the cropping systems in various agro-ecologies. Traditional analyses of such data focus on yield and yield components at the local level. Given limitation in their scope, such analyses fail to provide the evidence needed to guide policy and good practice. In order to draw broader conclusions relevant to policy, the present work aimed at cross-country analysis to have an idea of the sustainability of the practices.

Sustainability is a very broad concept encompassing natural, social, and economic capital, and it would not be possible to present a single metric applicable to any situation [9,10,29]. As such, assessing the sustainability of the intercropping system in the conventional sustainability framework will be a daunting task. Instead, in this analysis we focused on comparing treatments using indicators of sustainable intensification, which have been reported to be more relevant to the system, such as the one studied below. Several indicators of SI are available in the literature, and these fall under five major domains, namely productivity, environmental sustainability, economic sustainability, social sustainability, and human wellbeing [29]. The main objective of this analysis was to assess the role of fertilizer use in cereal-pigeonpea intercropping in terms of agricultural productivity and economic indicators of SI, specifically yields, production risks, input use efficiency, and economic returns on smallholder farms. The results of this work are expected to lead to better understanding of the interactions between soil infertility and the livelihoods of smallholder farmers. This work is also aimed at providing practical solutions to address the key issue of soil fertility depletion through nutrient mining, which constrains land productivity and the well-being of farmers.

## 2. Materials and Methods

### 2.1 Study Areas and Treatments

The data came from on-farm trials conducted by three projects supported by Alliance for Green Revolution in Africa (AGRA) in Mozambique, Tanzania, and Kenya. Details of the trial sites are presented in Table 1. In terms of their farming systems and socio-economic context, the study areas are more or less homogenous. The farming system is dominated by the maize mixed farming system. This farming system covers 10% of the land area, including most of the plateau and highland areas of East Africa (including Kenya and Tanzania) and southern Africa (including Mozambique). It serves as the food basket, as well as the driver of agricultural growth and food security in the region. However, this farming system is currently in crisis due to several interacting factors, including decline in average farm sizes, low use of agricultural inputs, loss of nutrients and organic matter [2], greater poverty levels, and vulnerability to climate change [4,5].

**Table 1:** The sites covered by the projects, their dominant soils, and amounts of N and P fertilizer applied in the different countries.

Country	District/County	Agro-Ecology Dominant Soils ^‡^	Dominant Soils z	Fertilizer Dose ^†^	N Rate (kg ha^−1^)	P Rate (kg ha^−1^)
Mozambique	Barue	Sub-humid	Ferralsols	Full	73, 145	30
				Half	15	30
	Gorongosa	Sub-humid	Lixisols	Full	73	30
				Half	15	30
	Moatize	Sub-humid	Lixisols	Full	80	20
				Half	17	10
	Tsangano	Sub-humid	Lixisols	Full	80	20
				Half	34	17
	Angonia	Sub-humid	Lixisols	Full	68	35
				Half	34	17
	Vanduzi	Sub-humid	Lixisol	Full	73	30
				Half	15	30
	Susundenga	Semi-arid	Lixisol	Full	73	30
				Half	15	30
	Manica	Semi-arid	Phaeozems	Full	73	30
				Half	15	30
Tanzania	Arumeru	Sub-humid	Leptosols	Full	60	20
	Hai	Sub-humid	Nitisols	Full	60	20
	Kondoa	Semi-arid	Leptosols	Full	60	20
	Moshi	Sub-humid	Nitisols	Full	60	20
	Siha	Sub-humid	Nitisols	Full	60	20
Kenya	Makueni	Semi-arid	Ferralsols	Full	60	20
				Half	30	10

‡ The dominant soil types are strictly based on the harmonized soil atlas of Africa following the World Reference Base for Soil Resources (WRB) classification and correlation system. The United States Department of Agriculture (USDA) class equivalents are: Acrisols = *Ultisols*; Cambisols = *Inceptisols*; Ferralsols = *Oxisols*; Leptosols = *Entisols*; Lixisols = *Alfisols*; Nitisols = *Alfisols*.

† Full and Half represent the recommended and half or less than half of the recommended N and P fertilizer dosages.

In Mozambique, the trials were conducted during 2012–2014 in Angonia, Barue, Gorongosa, Moatize, and Tsangano districts. The sites are characterized by a single growing season and a unimodal rainfall pattern, followed by a long dry season that lasts for five months from December to April. In all the countries, the treatments included cereal-pigeonpea intercropping without fertilizer (used as the control in this analysis), sole cereal crop, and sole pigeonpea combined with fertilizer. The NPK fertilizer 12:24:12 was applied as a basal dressing and urea as top dressing. Sole pigeonpea receiving N (22.5 kg ha^−1^) and P (20 kg ha^−1^) was included on some sites. The N and P rates applied to the cereal varied with site (Table 1). On other sites, small N doses (≤50% the recommended rate) were applied to maize (Table 1). For brevity, these are referred to as “half” rate, while application rates exceeding this (≥50% of recommended) are referred to as “full”. Hybrid maize was intercropped with an improved medium-duration (150–200 days to maturity) pigeonpea variety (ICEAP 0557) of an indeterminate growth habit and water-limited yield potential of 3000 kg ha^−1^. The maize varieties were PAN 53 and PAN 67, which have a water-limited yield potential of 9000–10,000 kg ha^−1^.

The sites in Tanzania are characterized by a bi-modal annual rainfall cycle with the major rainy season (often called the long rains) occurring during March–June and the “short rains” during October–December. The trials were implemented during 2010–2012 in seven districts: Hai, Moshi, and Siha in Kilimanjaro region; Kondoa Districts in Dodoma region; and Arumeru District in Arusha region. The treatments included maize-pigeonpea intercropping and sole maize combined with the full rate of fertilizer, consisting of urea (60 kg N ha^−1^) and phosphorus fertilizer in the form of either diammonium phosphate (DAP) (20 kg P ha^−1^), Minjingu hyperphosphate (20 kg P ha^−1^), or Minjingu Mazao (20 kg P ha^−1^). Hybrid maize was intercropped with the same pigeonpea variety (ICEAP 0557) used in Mozambique. The maize variety used was PAN 691 with a water-limited yield potential of 8000 kg ha^−1^.

The trials in Kenya were conducted during 2012–2014 on farms spread across Kwa Kakulu and Kavingoni locations in Makueni County in the eastern semi-arid lowlands. The sites have bimodal rainfall, as in Tanzania. Makueni County is largely semi-arid and usually prone to frequent droughts. The treatments consisted of intercropping of sorghum (variety Gadam) with pigeonpea, sole sorghum, and sole pigeonpea. The intercrops received either half or full rate of the recommended fertilizer for sorghum. The N fertilizer was applied in the form of calcium ammonium nitrate (CAN), while the phosphorus fertilizer was applied in the form of DAP. The full rate of fertilizer consisted of 60 kg N ha^−1^ and 20 kg P ha^−1^, while the half rate was 30 kg N ha^−1^ and 10 kg P ha^−1^. An improved pigeonpea variety (Mbaazi 2) with a water-limited yield potential of 3200 kg ha^−1^ was used on all sites. This variety has an indeterminate growth habit, taking more than 220 days to mature. Planting date, population densities, planting methods, weeding, and all other agronomic practices were performed, as per extension recommendation for the specific study areas.

### 2.2 Study Design

SI relies on collaboration between researchers and farmers to develop locally appropriate agricultural technologies [10]. In that spirit, the design involved the mother-baby participatory trial concept, which systematically links a central “mother trial” managed by researchers to numerous farmer-managed “baby” trials [28]. The central mother trial tests a large number of best-bet technologies or varieties and is replicated within a site, whereas the baby trials test a smaller subset of technologies. This facilitates rigorous cross-checking of agronomic performance with farmers’ assessment. Thus, the approach also allows the testing of multiple technologies from which individual farmers co-learn and select the best-bet practices for their own use [28]. Each plot was 20 × 20 m (400 m^2^). Over the course of 3–4 years, the total number of mother demonstrations was 85 in Kenya, 430 in Tanzania, and 385 in Mozambique. During this period, the total number of farmers engaged in cereal-pigeonpea intercropping was estimated at 14,750 in Kenya, 18,000 in Tanzania, and 22,820 in Mozambique.

### 2.3 Statistical Analysis

In this analysis we focused on a few of the frequently cited SI indicators, for which information was available within the scope of the trials. These included yield, production risk, input use efficiency, and agricultural income, all of which have been cited as strong SI indicators [29]. As such, the analysis is exclusively based on productivity and economic indicators.

#### 2.3.1 Crop Yield

Among the SI indicator in the literature, crop yield is by far the most common one [29]. This analysis focused on cereal grain yield, stover biomass, and wood yield of pigeonpea across sites in each country. For all analyses, we used a linear mixed modelling framework because data were unbalanced in terms of number of treatments and sample sizes. Our focus was on comparison of cereal-pigeonpea intercropping that received the full recommended fertilizer (Intercrop + Full) with intercropping without fertilizer application (hereafter called “Intercrop-Unfertilized”). Wherever available, we also compared sole maize that received the full recommended fertilizer (Sole maize + Full) with Intercrop + Full or intercrop that received half of the recommended fertilizer for the cereal (Intercrop + Half). In all cases, we used the means and their 95% confidence limit for statistical inference. We interpreted treatments as significantly different from one another only if their 95% confidence limits were non-overlapping.

#### 2.3.2 Indicators Production Risk

In the context of SI, risk is generally measured as either production risk or perceived risk [29]. In this analysis we focused only on production risk. One of the most commonly used measures of risk is variability, usually indexed by the coefficient of variation (CV)—a larger CV reflecting more risk [29]. We calculated the CV in Intercrop + Full, Intercrop-Unfertilized, and Sole maize + Full wherever applicable. Another common measure of production risk is downside risk, which can be measured either as the number of years or sites for which returns or yields are below a target, i.e., deviations from a target. For this purpose, we estimated the probability of obtaining cereal yield below the target of 3000 kg ha^−1^ proposed [2]. We estimated this risk measure for maize yield across sites in Mozambique and Tanzania separately. We also combined all sites because the maize and pigeonpea varieties used across Mozambique and Tanzania were more or less the same. Then we plotted the cumulative probability distribution of yields to aid stochastic dominance analysis, a non-parametric risk analysis tool often used in decision theory. A cumulative distribution function of one treatment is said to dominate another if the first order of its distribution lies entirely to the right of the cumulative distribution of the other treatment.

#### 2.3.3 Indicators of Input Efficiency

Input use efficiency is the other commonly proposed indicator of SI [9]. Among the many metrics of input efficiency, partial factor productivity (PFP) is the most commonly used SI indicator [8]. Therefore, we calculated the PFP of N (PFPN) and P (PFPP) fertilizers applied to the cereal crop. PFP is measured in kg grain per kg of applied N or P. We also assessed the agronomic use efficiency (AE) of N and P (i.e., AEN and AEP), measured in kg grain increase per kg of applied N or P. The AE is an integrated index of nutrient recovery efficiency and closely reflects the impact of the applied N or P fertilizer on grain yield. We focused the comparison of input use efficiency on those treatments where cereals received N and P inputs, i.e., Intercrop + Full and Intercrop + Half with Sole maize + Full.

#### 2.3.4 Indicators of Agricultural Income

Several metrics of agricultural income are used in the SI literature, but the most common one is the benefit cost ratio (BCR) [29]. We calculated BCR using data on input prices (costs of fertilizer, labor, and transport) and outputs (farm-gate of maize and pigeonpea) acquired through market surveys in each country. We calculated BCR as the ratio of total returns to total costs for each treatment at each mother trial. As in the analysis of yields, we used a linear mixed modelling framework to estimate the mean BCR and its 95% confidence limits for each treatment. For the adoption of technologies, the break-even point (BCR = 1) is often not sufficiently attractive, but BCR significantly larger than 1 is generally considered to be profitable. Therefore, a treatment was deemed profitable when BCR was significantly greater than 1, as judged by the width of the 95% confidence limit.

Acceptability of fertilizer by farmers is best judged by the marginal rates of return (MRR), an approach to maximize profit [30]. Therefore, MRR was computed as the ratio of the marginal benefit (i.e., the change in net benefits) divided by the marginal cost (i.e., the change in costs) relative to the intercrop without fertilizer. As a rule of thumb an MRR less than 50% is considered low and unacceptable to farmers; a higher cut-off value (e.g., MRR > 100%) has been recommended if the technology involves significant change from current farmer practices [30]. Since the application of fertilizer to the intercrop does not represent significant adjustment to current farmer practice, MRR of 50% was set as an acceptable minimum in this analysis.

Since pigeonpea can be managed as a perennial crop in cereal cropping systems [15], investments made in the first year are expected to provide benefits 2–4 years later. Therefore, we estimated the net present value (NPV) by discounting and summing the net benefit for each year. The major assumptions in the NPV calculation were the time horizon (t) and discount rate. In this analysis, a 5-year time horizon was considered reasonable. For discount rates, NPV analyses typically use loan interest rates which are set by national banks. During the project implementation period, interest rates were 14–17% in Kenya, 15–17% in Tanzania, and 14–19% in Mozambique [31]. Therefore, we used the country-specific discount rate based on the loan rate as a proxy for the years 2013 and 2014, which were 17% for Kenya, 16% for Tanzania, and 15% for Mozambique. If the NPV is positive, the benefits outweigh the costs, and the investment will generate a profit over time. We also conducted sensitivity analysis to detect the influence of volatility in output prices on financial returns. We computed NPV, assuming 10 and 20% fall or rise in maize and pigeonpea prices while holding cost of inputs constant. We held the cost of inputs constant and varied the crop prices because crop prices usually fluctuate widely in a year while input prices often remain constant. We then compared the outcome with a “no change” scenario in prices. In order to reduce clutter, we only focused the sensitivity analysis on intercropping + full fertilizer, which was found to be the best practice.

## 3. Results

### 3.1 Cereal Yields, Variability and Risk

**Figure 1:**
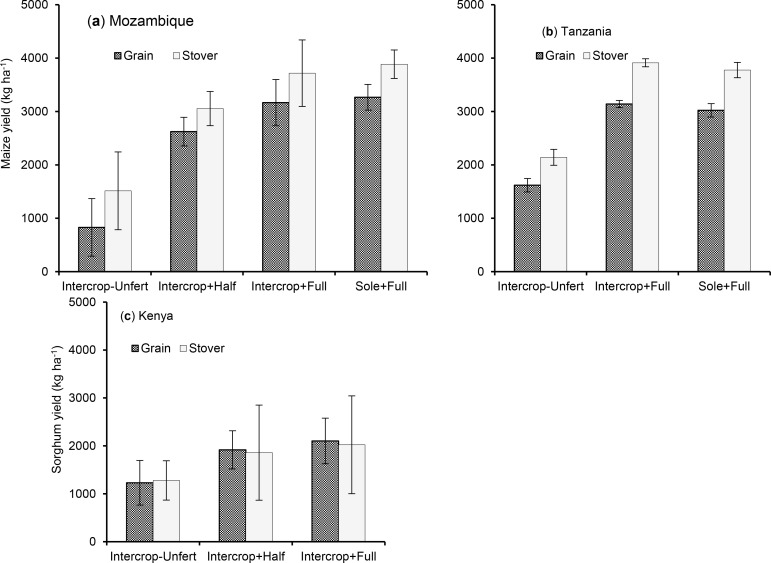
Variation in cereal grain and stover yields with fertilizer application in cereal-pigeonpea intercropping in Mozambique (**a**), Tanzania (**b**) and Kenya (**c**). Intercrop-Unfert = unfertilized cereal-pigeonpea intercrop. Two treatments are deemed significantly di_erent when their 95% confidence limits (error bars) do not overlap.

**Figure 2:**
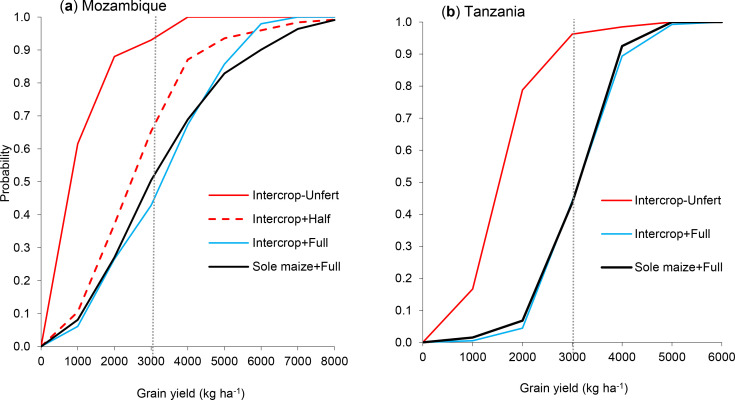
The probability distribution of maize yields in relation to the target yield of 3000 kg ha-1 in Mozambique (**a**) and Tanzania (**b**). Yields below the dotted vertical line indicate production risks.

Fertilizer use in intercropping (Intercrop + Full) reduced variability in cereal yields (CV) by 40–56% and increased yields by 55–294% over unfertilized intercrops (Intercrop-Unfertilized) across sites in the three countries (Table 2). Across the sites in Mozambique, maize grain yield was highest (3267 kg ha^−1^) in fully fertilized sole maize (Sole maize + Full) but its variability was also highest (CV = 108%). On the other hand, in the Intercrop + Full, the variability was lowest (CV = 47.6%), although the mean yield (3165 kg ha^−1^) was not significantly different from Sole maize + Full (Figure 1a). Yields recorded in Intercrop-Unfertilized (829 kg ha^−1^) were significantly lower than in Intercrop + Full, besides being more variable (CV = 107.9%) (Table 2). Across the sites in Tanzania, maize yield was highest (3130 kg ha^−1^) but its variability was lowest (CV = 22.2%) in Intercrop + Full. Yields were significantly higher than in Intercrop-Unfertilized (1625 kg ha^−1^) (Figure 1b), which had the highest variability (CV = 45.6%). The probability distribution of yields revealed first order stochastic dominance of Intercrop + Full over Intercrop-Unfertilized (Figure 2). Across the sites in Mozambique and Tanzania the probability of exceeding 3000 kg ha^−1^ was significantly higher in Intercrop + Full than in Intercrop-Unfertilized (Figure 2). When data were combined, the probability was higher (0.56) in Intercrop + Full compared to 0.04 in Intercrop-Unfertilized.

**Table 2:** Cereal and pigeonpea grain yields (in kg ha^—1^), their coe_cients of variation (CV in %), and percentage change in CV from the intercrop without fertilizer (Intercrop-Unfertilized).

Crop	Country	Treatment	Mean Yield	CV (%)	% Change in CV
Maize	Mozambique	Intercrop-Unfertilized	829	107.9	
		Intercrop + Full	3165 (282.1) ^*^	47.6	−55.9
		Intercrop + Half	2623 (216.6)	57.6	−46.7
		Sole maize + Full	3267 (294.3)	55.8	−48.3
	Tanzania	Intercrop-Unfertilized	1625	45.6	
		Intercrop + Full	3130 (92.6)	22.2	−51.4
		Sole maize + Full	3021 (85.9)	24.2	−47.0
Phosphorus	Mozambique	Intercrop-Unfertilized	1230	59.6	
		Intercrop + Full	2102 (71.0)	35.6	−40.2
		Intercrop + Half	1917 (55.9)	32.7	−45.1
		Intercrop-Unfertilized	252	93.7	
		Intercrop + Full	1384 (449.5)	77.7	−16.6
		Intercrop + Half	1307 (419.1)	88.0	−5.5
		Sole Pigeonpea + Full	3187 (1164.7)	43.4	−53.4
	Tanzania	Intercrop-Unfertilized	346	71.4	
		Intercrop + Full	994 (187.2)	62.1	−13.0
	Kenya	Intercrop-Unfertilized	904	60.6	
		Intercrop + Full	1191 (31.7)	29.0	−52.2
		Intercrop + Half	970 (7.3)	38.3	−36.8

* Figures in parentheses are percentage yield increase over the Intercrop-Unfertilized.

In Kenya, sorghum yield was significantly higher (2102 kg ha^−1^) and variability was lower (CV = 35.6%) in Intercrop + Full (Figure 1c) compared to Intercrop-Unfertilized (yield = 1230 kg ha^−1^ and CV = 59.6%). Maize and sorghum stover yields followed the same pattern as grain yield in all countries, with the highest being in Intercrop + Full (Figure 1).

### 3.2 Pigeonpea Yields and Variability

**Figure 3:**
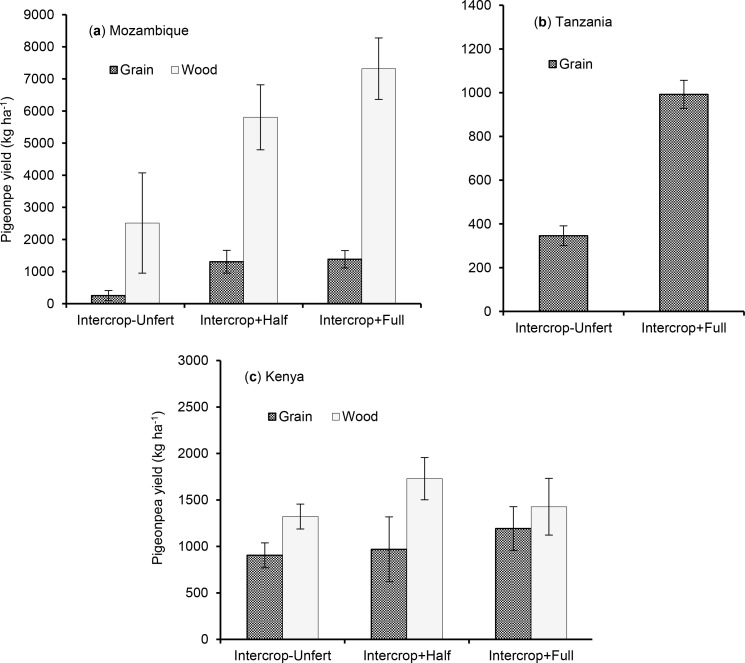
Variation in pigeonpea grain and wood yield with fertilizer rate in cereal-pigeonpea intercropping in Mozambique (**a**), Tanzania (**b**) and Kenya (**c**). Intercrop-Unfert = unfertilized cereal-pigeonpea intercrop. Vertical bars represent the 95% confidence limits. In Tanzania wood biomass was not determined. Yields of sole pigeonpea were excluded from figures because these were available only for some sites

Pigeonpea yields were 31–449% higher in Intercrop + Full over Intercrop-Unfertilized (Table 2). Fertilizer use in intercropping reduced variability in yields of pigeonpea by 5–52% over Intercrop-Unfertilized. Across the sites in Mozambique where sole pigeonpea received N and P fertilizer, grain yield was 3187 kg ha^−1^ compared to 1384 kg ha^−1^ in Intercrop + Full and 252 kg ha^−1^ in Intercrop-Unfertilized (Table 2). In Kenya, Intercrop + Full gave pigeonpea grain yield of 1191 kg ha^−1^ compared to 904 kg ha^−1^ in Intercrop-Unfertilized and 680 kg ha^−1^ in sole pigeonpea grown without fertilizer. Intercrop + Full fertilizer consistently gave the highest wood yields, ranging from 1729 kg^−1^ ha across sites in Kenya to 7318 kg ha^−1^ in Mozambique (Figure 3a,c). Wood yield measurements were unavailable for sites in Tanzania (Figure 3c).

### 3.3 Input Use Effciency

The partial factor productivities of N and P were consistently higher (by up to 50%) in Intercrop + Full than Sole maize + Full (Table 3). Across sites in Mozambique partial factor productivities of N and P were significantly higher in Intercrop + Half than in Sole maize + Full. AEN was 227% higher in Intercrop + Full than in Sole maize + Full fertilizer. On the sites in Tanzania, Intercrop + Full and Sole maize + Full did not significantly differ in both partial factor productivity and agronomic use effciency of N, but the former consistently had higher values (Table 3). When data were combined across sites in Mozambique and Tanzania, AEN and AEP were 37% and 50% higher in Intercrop + Full than in Sole maize + Full (Table 3). In Kenya, AEN was 57% higher in sorghum-pigeonpea intercropping that received half of the recommended fertilizer than fully fertilized sorghum-pigeonpea intercropping. AEP also followed the same trend as AEN (Table 3).

**Table 3:** Partial factor productivity (PFP) and agronomic efficiency (AE) of N and P in sole cereal and in intercropping with pigeonpea with either half or the full dose of fertilizer recommended for the cereal in Mozambique, Tanzania, and Kenya.

Nutrient	Country	Treatment	PartialFactorProductivity^*^	Agronomic Efficiency ^**^
Nitrogen	Mozambique	Intercrop + Half	148.3 (137.7–158.9)	47.1 (38.0–56.2)
		Intercrop + Full	42.9 (26.1–59.7)	16.6 (0.4–56.2)
		Sole maize + Full	34.6 (26.7–42.5)	14.4 (7.6–21.2)
	Tanzania	Intercrop + Full	52.3 (51.2–53.5)	25.5 (24.0–26.9)
		Sole maize + Full	50.4 (48.4–52.3)	25.1 (22.4–27.8)
	Kenya	Intercrop + Half	63.9 (54.2–73.6)	22.9 (13.3–32.5)
		Intercrop + Full	35.0 (25.3–44.8)	14.5 (5.0–24.1)
Phosphorus	Mozambique	Intercrop + Half	145.7 (130.9–160.4)	61.4 (46.7–76.1)
		Intercrop + Full	123.9 (100.4–147.4)	48.7 (22.5–75.0)
		Sole maize + Full	84.8 (73.7–95.8)	35.7 (24.7–46.7)
	Tanzania	Intercrop + Full	157.0 (153.6–160.4)	76.4 (72.1–80.7)
		Sole maize + Full	151.1 (145.2–157.0)	75.3 (67.3–83.3)
	Kenya	Intercrop + Half	191.7 (162.5–220.9)	68.7 (39.9–97.4)
		Intercrop + Full	105.1 (75.9–134.3)	43.6 (14.9–72.3)

* Partial factor productivity is given in kg grain kg^−1^ of applied N or P.

** Agronomic efficiency is given in kg grain increase kg^−1^ of applied N or P. Figures in parentheses represent 95% confidence limits

### 3.4 Profitability of Fertilizer Use

The benefit–cost ratios (BCR) were significantly larger than unity in Intercrop + Full compared to Intercrop-Unfertilized (Table 4). Across sites in Mozambique Intercrop + Half was more profitable (BCR = 1.4 and MRR = 145) than Intercrop + Full (BCR = 1.1 and MRR = 38.7). With a BCR of 0.7, Sole maize + Full was as unprofitable as Intercrop-Unfertilized (Table 4). With Intercrop + Full, MRR was 178% in Tanzania, meaning that farmers in the study sites can expect to obtain $2.78 for every $1 investment in purchasing and applying fertilizer in the intercropping. In Kenya, Intercrop + Half was slightly more profitable (MRR = 116) than Intercrop + Full (MRR = 82).

**Table 4:** Benefit–cost ratios (BCR), net present values (NPV), and marginal rates of return (MRR) in intercropping, with and without fertilizer.

Country	Treatment	BCR	MRR	NPV-5 Year
Mozambique	Intercrop-Unfertilized	0.7 (0.3–1.2) *	NA	−301.1 (−796.3–194.1)
	Intercrop + Full	1.1 (1.0–1.3) ^*^	38.7 ^§^	377.3 (^−^6.7–761.2)
	Intercrop + Half	1.4 (1.2–1.5)	145.2	733.5 (338.2–1128.8)
	Sole maize + Full	0.7 (0.6–0.8) ^*^	33.7 ^§^	^−^937.7 (^−^1092.7–^−^782.6)
Tanzania	Intercrop-Unfertilized	1.1 (1.0–1.1) ^*^	NA	67.2 (^−^55.5–189.9)
	Intercrop + Full	1.5 (1.4–1.6)	177.7	1018.9 (906.9–1130.9)
	Sole maize + Full	1.4 (1.3–1.4)	290.2	574.4 (488.8–659.9)
Kenya	Intercrop-Unfertilized	1.3 (0.9–1.6) ^*^	NA	593.3 (^−^156.1–1342.6)
	Intercrop + Full	1.4 (1.2–1.7)	82.1	1192.3 (491.8–1892.9)
	Intercrop + Half	1.4 (1.1–1.7)	116.5	1019.7 (350.6–1688.8)

The figures in parenthesis are 95% confidence limits.

* When the 95% confidence limit encompasses 1, BCR is not significantly different from 1, and thus the intervention is unprofitable. NA = not applicable. ^§^ MRR is below the threshold of 50%.

In terms of net present values (NPV), Intercrop + Full was more profitable than Intercrop-Unfertilized across all countries (Table 4). The NPV was more than 2 times higher in Intercrop + Full compared with Intercrop-Unfertilized in Tanzania and Mozambique. In Kenya NPV was 101% higher in Intercrop + Full compared with Intercrop-Unfertilized (Table 4). When all sites were considered, there was 86% probability of producing maize and pigeonpea profitably (NPV > 0) in Intercrop + Full. On the other hand, in Intercrop–Fertilizer there was only 47% probability of producing maize and pigeonpea profitably.

**Figure 4:**
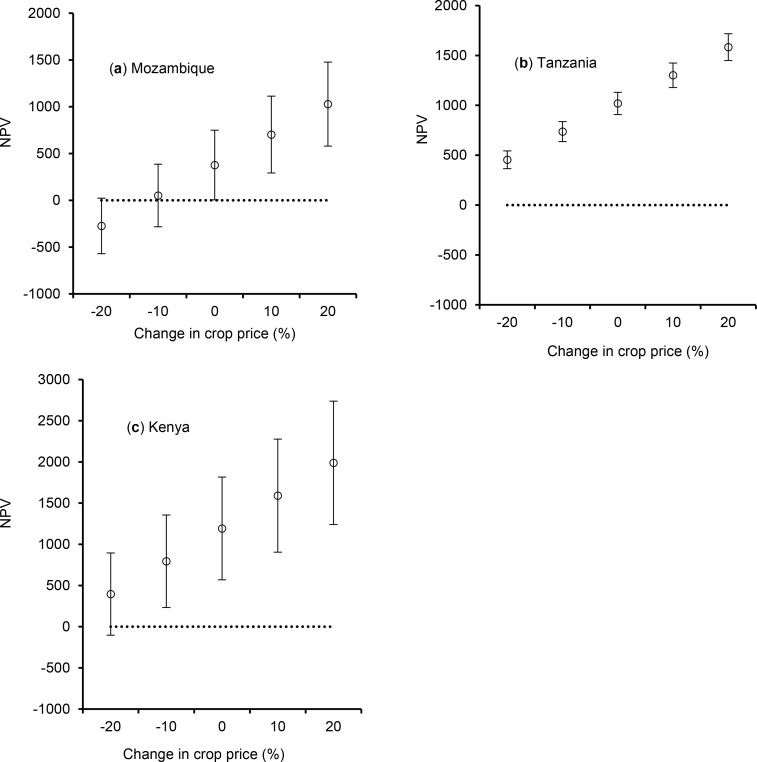
E_ect of price volatility on net present value (NPV) of cereal-pigeonpea intercropping with the full fertilizer rate in Mozambique (**a**), Tanzania (**b**) and Kenya (**c**). When the 95% confidence limit encompasses the dotted horizontal line, intercropping becomes unprofitable.

According to the sensitivity analysis, an increase in maize and pigeonpea prices by 10–20% will not significantly increase the profitability (i.e., NPV) of the intercrop in Mozambique and Kenya (Figure 3). A fall in prices by 10–20% will make intercropping unprofitable in Mozambique. On the other hand only a 20% fall in prices will make intercropping unprofitable in Kenya. In Tanzania the 95% confidence limits do not overlap, indicating that intercropping will continue to be profitable even with a 20% fall in crop prices (Figure 4). Increases in maize and pigeonpea prices by 10% will also significantly increase NPV.

## 4. Discussion

All the SI indicators assessed indicated that intercropping combined with the application of small amounts of fertilizer to the cereals is superior to intercropping without fertilizer. Indeed, in the absence of fertilizer inputs, intercropping maize with pigeonpea gives only modest yields. These findings are consistent with findings from earlier studies in Zimbabwe [26,32] and Tanzania [20]. These earlier studies have also reported depression in yields of maize in intercropping relative to monoculture maize. This has been attributed to competition between maize and pigeonpea for soil nutrients and water [20]. Our results also suggest that pigeonpea yields are suppressed in intercropping when fertilizer is not applied to the maize crop. This is consistent with reports from earlier studies [20] that showed strong competition from the maize crop, thus suppressing grain yields of the pigeonpea by as much as 33%. The increase in grain of pigeonpea is probably because the fertilizer applied to maize may have benefitted the pigeonpea, since P fertilizer is crucial for growth and N fixation in legumes. In Mozambique, where N and P were added to sole pigeonpea, grain yields increased by 3–4 times over the unfertilized intercrop. Similarly, in Tanzania the addition of N and P fertilizers has been shown to increase pigeonpea biomass yield by 239% relative to the unfertilized intercropping treatment [33].

Higher yields with fertilizer use in intercrops implies that farmers could produce more without putting extra land under cultivation. The increase in yields is expected to diversify food and income sources for household. With farmgate prices often 3–4 times more than maize, market opportunities for pigeonpea are greater than those for maize in the study areas. As such, sales of pigeonpea are likely to contribute to farmers’ ability to purchase the small quantities of fertilizers required seasonally. The additional benefit of intercropping is the wood biomass from pigeonpea harvest, which can be used as a domestic energy source [21]. When pigeonpea production is integrated with energy-saving stoves, it can also reduce the frequency of buying and collecting fuel-wood [21]. This could free some time for women in rural areas who traditionally do the task of firewood collection for the family. The increase in crop residues (maize or sorghum stover and pigeonpea wood) due to addition of fertilizer can also add significant benefits to the farm economy. Crop residues are valuable sources of animal feed and soil organic matter. For example, stover is increasingly becoming an important source of soil cover in conservation agriculture (CA). In the absence of fertilizer, average stover yields were well below the threshold of 3.0 t ha^−1^ required for good soil cover. Stover yields less than this will not provide the 30% soil cover required to implement CA successfully [34]. There is also the pigeonpea litterfall,

which although not quantified in this study, could be significant in its contribution to soil fertility improvement. This could, in the short and medium term, break the cycle of land degradation and its associated social deprivation issues [35].

Animportantbenefitofapplyingsmallamountsoffertilizersinintercropsisthemoreefficientuseof the applied N and P fertilizers compared to sole maize. This is probably because intercropping increases availability of applied nutrients and improves nutrient use efficiency by associated cereals [20,36]. Fertilizer use is not only essential to increase crop yields but also replenish the nutrients removed by crop harvest, and thus sustaining productivity due to a more efficient use of fertilizers. In many parts of East Africa, the soils are P deficient [2] and its omission has been shown to depress the attainable yields of maize by 1000–1700 kg ha^−1^ year^−1^ [37]. Without fertilizer application and other good agronomic practices, intercropping pigeonpea with high yielding varieties of maize is likely to be unsustainable.

Our analyses also indicate that the strategic application of small amounts of fertilizers (e.g., Intercrop + Half) is profitable in the intercropping systems in the majority of cases, except where 10–20% fall in maize and pigeonpea prices makes intercropping unprofitable with application of the fertilizer recommended for maize. This was the case in Mozambique, where higher input prices (compared to Tanzania and Kenya) reduced profitability. The positive NPV values in all countries indicate that overall the benefits of fertilizer use outweigh the costs and the investment in fertilizer in cereal-pigeonpea intercropping is profitable. As such, intercropping pigeonpea with cereals can be a good source of cash for resource-poor and vulnerable households. Good markets exist for pigeonpea products from Africa, and the demand for processed pigeonpea products outstrips supply by over 30% [16]. For example, the annual pigeonpea grain exports to India in the last decade recorded a high of about 2500 tons against an estimated demand of 15,000–20,000 tons [23]. Due to the high demand, farmers in the study sites were receiving farmgate prices for their pigeonpea crops that were 3–4 times that of maize. Given this market potential, application of the recommended dose of fertilizer in intercropping may continue to be profitable even if maize and pigeonpea prices fall by as much as 20%.

Another important finding from this analysis is the lower yield variability where the intercrop was amended with fertilizer. Reduced variability implies reduced risk of income loss, while higher benefit–cost ratio results in higher net income. The economic analysis suggests the benefit–cost ratios vary with sites, with some being profitable at half the recommended nitrogen and phosphorus application ratesof the nationalsystems, which are generally 60and 20 kgha^−1^, respectively. This should help farmers purchase the small amounts of fertilizers they need to improve the fertility of their soils and raise its productivity.

Overall, these results indicate that farmers are better off planting pigeonpea in their maize fields, wherever it is suitable, than simply growing sole maize with the recommended fertilizer rate. In contrast with the emphasis on cereal monocultures generally by some development agencies, our results, and those of the growing body of literature [8,17,38–40], suggest that intercropping can play a central role in the efforts to achieve climate-smart agriculture. For example, in Malawi and Mozambique, maize-pigeonpea intercropping without fertilizer application has been demonstrated to reduce the risk of crop failure and improve profitability [8,17,38,39]. Using historical rainfall records and simulated yield in Malawi, Snapp et al. [40] showed that intercropping pigeonpea with maize can meet the household food needs (calories and proteins) in 73–100% of the years across variable rainfall patterns. According to Kamanga et al. [38], maize intercropped with pigeonpea was less risky for resource-poor farmers compared to fully fertilized maize in central Malawi. Pigeonpea is well adapted to semi-arid conditions, and tolerant to many environmental stresses [16]. These are important traits for enhancing the resiliency of smallholder farmers to the growing challenge of climate change and climate variability. However, there are challenges associated with *Fusarium* wilt, which was noticeable among pigeonpea stands of even supposedly tolerant varieties.

Intercropping of cereal with pigeonpea can benefit not only the resource-endowed farmers but also very poor households. For example, in central Malawi, intercropping maize with pigeonpea had consistently positive returns across all farmer resource groups [38]. Similarly, the most vulnerable households in southern Malawi are better off intercropping pigeonpea with maize than growing sole maize with the recommended fertilizer [39]. This can lead to higher productivity, as is evident from our study as well, which could in turn reduce the unit cost of production. This will not only increase productivity and profitability but it will also accrue other benefits as it can abate the effects of land degradation, which poses serious social, economic, and environmental problems in SSA [7]. Recent analysis of SI indicators in Malawi [41] also indicate that social and human capacity building were superior for pigeonpea maize intercropping rather than fully-fertilized maize monoculture, notably in terms of dietary diversity, food security, and farmer preferences.

The challenge has been lack of knowledge and the poor access to fertilizer and seeds of improved varieties by African farmers. Access to improved varieties of legumes is limited because production of legume seed is apparently not an attractive venture for the existing seed companies in the study locations. Policies on input subsidies also rarely provide for improved legume seed. If input subsidy programs could broaden their scope to include leguminous crops like pigeonpea, they would be better able to support the poorest households. There is also an urgent need to invest in training farmers on good agronomic practices that increase the use efficiency of inorganic fertilizers in maize-pigeonpea intercropping systems.

## 5. Conclusions

The main conclusion from the present analyses is that the strategic application of small amounts of fertilizers (15–30 kg N ha^−1^ and 10–20 kg P ha^−1^) along with good agronomic practices is necessary for cereal-pigeonpea intercropping to be sustainable and financially profitable in the semiarid regions of ESA. Application of fertilizer would continue to be profitable even if cereal and pigeonpea prices fall by as much as 20%. However, without fertilizer use, intercropping alone is likely to suppress pigeonpea yields, while also increasing nutrient mining when high yielding pigeonpea varieties are used. Therefore, it is important to encourage farmers to use the appropriate rates, especially of N and P fertilizers or manure, to the cereal-pigeonpea intercropping system. Application of small amounts of fertilizers, about half the levels recommended by the national research organization (for maize yield targets of 3 t ha^−1^ or more), may also help stabilize yields across seasons and minimize risks of crop loss to the farmers in the event of sub-optimal cropping seasons. However, there is a need to establish the levels more precisely through elaborate trials that use fertilizer response curves. Given the generally high cost of fertilizers in SSA, subsidies that are being promoted in several countries could be designed to target leguminous crops that have the potential to sustainably improve food security in the drier agro-ecologies and build more climate-smart and resilient production systems, such as pigeonpea. An important caution to note is that when pigeonpea intercropping is scaled-up, diseases (e.g., *Fusarium* wilt) and pests may threaten its production and productivity. Therefore, there is a need for continuous breeding for pest resistance and high-yielding pigeonpea varieties for areas that are currently under the crop and others that could potentially be brought under it. Scaling up the production of pigeonpea intercropping could contribute significantly to household firewood and energy supply, thus reducing pressure on forests and woodlands in the drylands.
